# The London Hospitals

**Published:** 1923-12

**Authors:** 


### THE LONDON HOSPITALS.

### ANALYSIS OF THE STATISTICAL REPORT.

IN our November issue we printed a necessarily hasty summary of the contents of the Statistical Report of King Edward's Fund on the Income and Expenditure of the One Hundred and Thirteen London Hospitals for the year 1922. The more carefully and critically this Report is examined the more obvious it becomes that even the hospital expert can learn a good deal from its carefully compiled pages and its masses of statistics. Not only is there a valuable Prefatory Note, but there are four tabulated statements respecting the whole body of London hospitals, 113 in number, arranged alphabetically, which give particulars of accommodation and work, income, expenditure and cost of working, and a further section, covering 20 pages, consisting of tables of the average cost per occupied bed, analysed into 5 principal classes of expenditure, and of the cost, similarly analysed, per out-patient attendance, of 79 of the 113 hospitals, sorted into 10 groups, each group consisting of institutions of a similar kind. The reason that these tables include only 79 of the 113 is that 34 institutions are of a heterogeneous character.

### The Promise of the Future.

To the ordinary reader, who feels an interest in hospitals and would rejoice in their prosperity, who contributes according to his means to their support, but takes no active part in the administration of any one of them, the most interesting part of the Report is the Prefatory Note. Speaking of the state of the hospitals in 1922 as compared with 1913, this note shows that the beds available had increased in the 10 years, that the average number regularly occupied was also greater though not in proportion, and that the number of in-patients treated was disproportionally higher. On the other hand, out-patients were fewer in 1922, and yet the number of their attendances had greatly increased. Turning to finance, we are informed that, apart from the sum voted by Parliament, the proceeds of the Combined Appeal and other special distributions, the income of the hospitals in this decade had increased from £1,470,000 to £2,398,000. Taken by itself that is very satisfactory, but it assumes a very different complexion when viewed in relation to expenditure, which had grown from £1,192,000 to £2,572,000. Happily, the sombreness of the picture is relieved by the fact that, high though the flood of expenditure had risen, the tide had in fact turned in 1921, when the figure was £8,000 lower than in 1920, and in 1922 a further subsidence, of no less than £204,000, took place. Now when we see that this one year's reduction in expenditure exceeds in amount the aggregate deficiency of all the 113 hospitals during the same period, there is reasonable ground for the hope that the end of the current year will reveal a still further substantial fall, which, with income at least maintaining its 1922 level, will terminate the hospital financial crisis which has caused such widespread concern.

### The Value of Statistics.

In the earlier of these Reports—of which this is the fifteenth—the statistical tables dealt with work and expenditure only, and the hospitals were grouped in classes—Hospitals for Women, Ophthalmic Hospitals, and so on. This is an admirable system—by no means original, of course, as Burdett's Hospitals and Charities had adopted it before the foundation of the King's Fund—enabling the hospital manager to realise at its maximum the help that statistics can afford him. After awhile fresh tables of work and expenditure were introduced, in which 100 hospitals or so of heterogeneous character were assembled in alphabetical order. More recently a table respecting income, in which the hospitals were also arranged in that way, was added, presumably because the study of income, its fluctuations, the proportion which each class thereof bears to the total and so on, has an undoubted bearing on the consideration of expenditure.

### Comparative Grouping.

The declared object of the Fund in preparing these tables being to compare the figures of one hospital with those of another, and with the average of those hospitals with which it is most comparable, we are satisfied that no system could have been devised so effective for the purpose as that originally chosen. That the Fund should, in the later tables, which now comprise Sections I. to IV., pp. 16 to 43, have abandoned the system of segregating the 113 institutions into small clusters or family groups, in favour of jumbling them all together in one huge table, simply disposing them in alphabetical order, seems to us quite unaccountable. But even if it had been deemed that an alphabetical arrangement was preferable, we certainly should have thought that the disadvantage of having some tables on one plan and some on another would have been perceived and avoided. As we have said, the grouping system is incomparably superior to the alphabetical arrangement, and we believe that the exact significance of all the facts respecting the accommodation, work, and finance of the hospitals, viewed both individually and in their relation to each other, will never be readily comprehended until, either in this report or some other, tables are forthcoming year by year exhibiting the data contained in this report of accommodation, work, income, expenditure and average cost per bed, of the hospitsla grouped as in Section V. We also think that in the groups themselves it would be much better to marshal the hospitals in order of size so as to bring each one into juxtaposition with those nearest to it in dimensions, and so make the best of the " most comparable " theory, than to follow the present alphabetical arrangement, which, however useful in a collection of 113 institutions, is quite unnecessary in a parcel of 14, the greatest number contained in any one group. This, however, is a matter of comparatively minor importance.

